# Modeling the Future of a Wild Edible Fern Under Climate Change: Distribution and Cultivation Zones of *Pteridium aquilinum* var. *latiusculum* in the Dadu–Min River Region

**DOI:** 10.3390/plants14142123

**Published:** 2025-07-09

**Authors:** Yi Huang, Jingtian Yang, Guanghua Zhao, Zixi Shama, Qingsong Ge, Yang Yang, Jian Yang

**Affiliations:** 1 Sichuan Provincial Forest and Grassland Key Laboratory of Alpine Grassland Conservation and Utilization of Tibetan Plateau, College of Grassland Resources, Southwest Minzu University, Chengdu 610041, China; 110055@mtc.edu.cn (Y.H.); smuyangyang@utibet.edu.cn (Y.Y.); 2 Key Laboratory of Biodiversity and Environment on the Qinghai-Tibetan Plateau, Ministry of Education, School of Ecology and Environment, Tibet University, Lhasa 850000, China; geqingsong@stu.utibet.edu.cn; 3 Ecological Security and Protection Key Laboratory of Sichuan Province, Mianyang Normal University, Mianyang 621000, China; yjtdc@mtc.edu.cn (J.Y.); shamazixi@mnu.cn (Z.S.); 4 School of Life Science, South China Normal University, Guangzhou 510631, China; 2024010259@m.scnu.edu.cn

**Keywords:** climate change, cultivation areas, ensemble models, potential distribution, *Pteridium aquilinum* var. *latiusculum*

## Abstract

Under the pressures of global climate change, the sustainable management of plant resources in alpine gorge regions faces severe challenges. *P. aquilinum* var. *latiusculum* is widely harvested and utilized by residents in the upper reaches of the Dadu River–Min River basin due to its high edible and medicinal value. This study employed ensemble models to simulate the potential distribution of *P. aquilinum* var. *latiusculum* in this region, predicting the impacts of future climate change on its distribution, the centroid migration of suitable habitats, and niche dynamics. A production dynamics model was also constructed to identify current and future potential cultivation areas by integrating ecological suitability and nutritional component synergies. The results show that current high-suitability areas and core cultivation zones of *P. aquilinum* var. *latiusculum* are predominantly distributed in patchy, fragmented patterns across the Wenchuan, Li, Mao, Luding, and Xiaojin Counties and Kangding City. Under climate change, the “mountain-top trap effect” drives a significant increase in high-suitability areas and core cultivation zones, while moderate-to-low-suitability areas and marginal cultivation zones decrease substantially. Meanwhile, suitable habitats and cultivation areas exhibit a northward migration trend toward higher latitudes. The most significant changes in suitable area and cultivation zone extent, as well as the most pronounced niche shifts, occur under high-emission climate scenarios. This research facilitates the development of suitability-based management strategies for *P. aquilinum* var. *latiusculum* in the study region and provides scientific references for the sustainable utilization of montane plant resources in the face of climate change.

## 1. Introduction

The utilization of plant resources under climate change has long been a focal area of global concern, attracting the attention of researchers across various disciplines, including geography, ecology, and archeology [1,2,3,4,5]. Existing studies indicate that among numerous influencing factors, non-climatic factors dominate short-term biological changes in plants. In contrast, climate change remains the primary driver affecting plant growth, development, and suitable distribution [5,6]. Herbivorous plant resources, as a unique category, are closely intertwined with human development [1,7,8]. The nutritional components of herbivorous plants form the basis for their edibility, with rich nutrients and favorable flavors serving as critical prerequisites for their selection by local communities [9,10]. Research indicates that climate change will affect the habitats of herbivorous plants, potentially leading to significant shifts and migrations in their spatial distribution, while altering the quantity and quality of their nutritional components [11,12,13,14,15]. Given the long-term persistence of climate change, scientifically evaluating its future impacts on the distribution of herbivorous plants is of great value for biodiversity conservation and sustainable ecosystem management.

Species distribution models (SDMs) utilize species occurrence records and environmental data (e.g., climate, topography) to construct species niches through specific algorithms, project them onto the entire study area, and ultimately display suitable habitats or potential distribution areas as probability maps [16,17,18,19]. Over the past decade, using SDMs and climate change scenarios to predict plant species’ suitable habitats has become a hotspot in global change ecology and plant ecology research [20,21,22]. SDMs encompass diverse types with significant methodological differences. The Biomod2(V4.25) platform in R software allows users to customize ensemble models by freely combining algorithms for target species [19,23,24]. While inherent flaws in individual models are unavoidable, optimal simulation results can be achieved by assigning weights to each model within the ensemble [25,26].

*Pteridium aquilinum* var. *latiusculum*, renowned as the “king of wild vegetables,” is also called Jixiangcai (lucky vegetable) or Longtoucai (dragon-head vegetable). Belonging to the genus *Pteridium* within the family *Pteridaceae*, this herbaceous plant thrives in sunny areas, and is primarily found in sparse coniferous and mixed broad-leaved forests. It is harvested when its young stems and leaves are tender and covered with white villi [27,28]. Fresh *P. aquilinum* var. *latiusculum* is rich in nutrients, including protein, dietary fiber, and vitamins, and contains eight essential amino acids and multiple trace elements, making it a delicious, tender, and highly nutritious option [27,29]. Additionally, it is abundant in bioactive components, including terpenoids, steroids, organic acids, flavonoids, and polysaccharides, offering health benefits such as swelling reduction, sedation, nourishment, anti-inflammatory effects, heat-clearing properties, expectorant effects, antioxidant properties, and hypoglycemic effects through regular consumption [30,31,32,33]. It is noteworthy that moderate consumption of *P. aquilinum* var. *latiusculum* is safe, but excessive or long-term heavy consumption may pose health risks. This is because *P. aquilinum* var. *latiusculum* contains ptaquiloside, which may pose a carcinogenic risk, while oxalic acid and crude fiber may trigger indigestion or carry stone-related risks [29,32,33].

The upper reaches of the Dadu River–Min River basin are characterized by a dual attribute of “alpine gorge barrier” and “resource-constrained livelihoods” [34,35]. Due to topographical constraints and transportation barriers, traditional livelihoods still play a significant role in the daily lives of residents. Field surveys indicate that *P. aquilinum* var. *latiusculum* is widely utilized by residents in this region; it is primarily collected and consumed as a food source. From April to May each year, locals harvest their young shoots as wild vegetables, which are processed through stir-frying, blanching, or cold dressing (Figure S1). Relevant studies indicate that some wild herbivorous plants in this region are highly vulnerable to climate change [36,37]. Current research on *P. aquilinum* var. *latiusculum* primarily focuses on its chemical composition, plant physiology, and pharmacological effects [30,31,32,33,38], but the mechanisms of adaptation to climate change in the Dadu River–Min River basin remain unclear.

In summary, this study focuses on the upper reaches of the Dadu River–Min River basin and *P. aquilinum* var. *latiusculum* to achieve the following objectives: (1) predict changes in suitable habitat areas under different climate scenarios, (2) analyze niche dynamics of *P. aquilinum* var. *latiusculum* under future climate conditions, and (3) develop a suitability–productivity model to delineate potential cultivation areas. These results will provide a theoretical foundation for the sustainable utilization of *P. aquilinum* var. *latiusculum* in this region.

## 2. Results

### 2.1. Current Distribution Projections and Model Accuracy Validation

**Figure 1:**
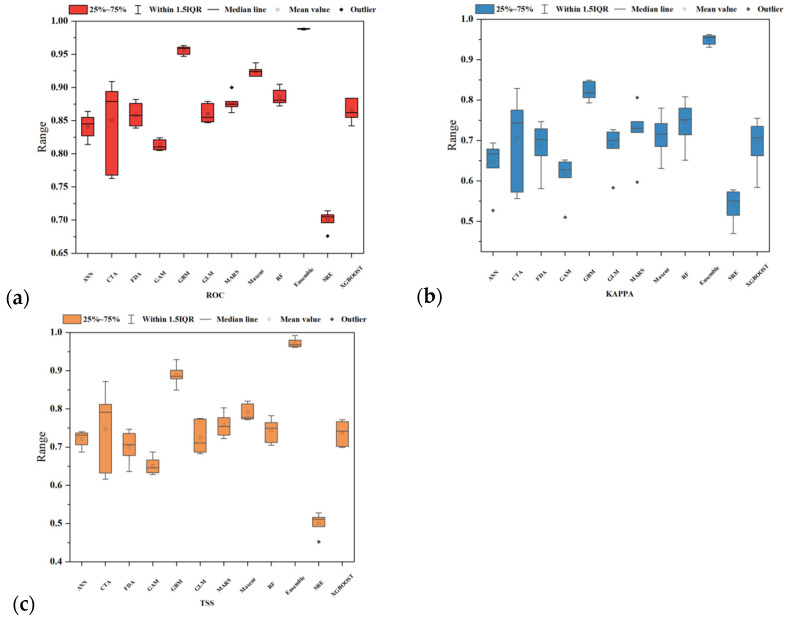
Evaluation of model accuracy using three metrics. (**a**) ROC; (**b**) KAPPA; (**c**) TSS.

The simulation results from each model for *P. aquilinum* var. *latiusculum* show that suitable areas are generally concentrated in the eastern, southern, and central parts of the upper Dadu River–Min River basin. While all models exhibited consistent overall trends, notable differences existed in their specific predictions (Figure S2). Evaluating model accuracy using valid metrics is critical for determining model reliability and applicability. This study used the “biomod_tuning” function to optimize model parameters, checking them in each iteration against selected metrics (ROC, Kappa, or TSS). Among single models, GBM, RF, and Maxent were ideal for predicting the potential spatial distribution of *P. aquilinum* var. *latiusculum*, whereas GAM, ANN, and SRE performed poorly (Figure 1). The ensemble model (“Ensemble”) demonstrated exceptional accuracy, with mean values of 0.972 for TSS, 0.998 for ROC, and 0.950 for KAPPA (Figure 1). The test results showed that Ensemble, constructed via weighted averaging, significantly outperformed other models in terms of precision. Thus, Ensemble provided the best fit and most reliable predictions.

### 2.2. The Current Potential Distribution of P. aquilinum var. latiusculum in the Upper Dadu River–Min River Basin

**Figure 2:**
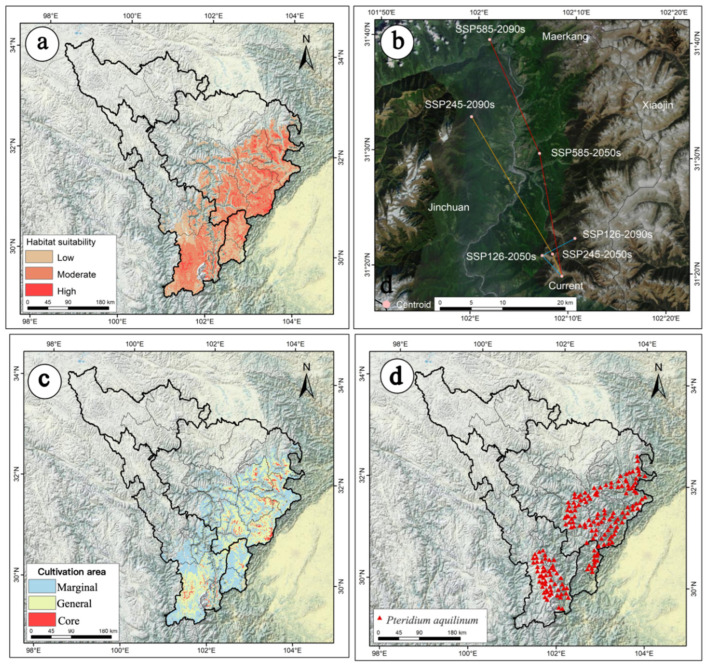
(**a**) The current potential distribution of *P. aquilinum* var. *latiusculum* in the upper Dadu River–Min River basin; (**b**) the migration trajectory of the habitat centroid for *P. aquilinum* var. *latiusculum* in the upper Dadu River–Min River basin; (**c**) the current potential cultivation areas of *P. aquilinum* var. *latiusculum* in the upper Dadu River–Min River basin; (**d**) distribution records of *P. aquilinum* var. *latiusculum* in the upper Dadu River–Min River basin.

The predictive model for *P. aquilinum* var. *latiusculum*’s potential suitable habitat exhibited excellent fitting performance. As shown in Figure 2a, the total area of suitable habitat was 6.23 × 10^4^ km^2^ (Table 1). High-suitability areas accounted for 0.36 × 10^4^ km^2^ (5.78% of total suitable habitat), primarily distributed in patchy, fragmented patterns across the Wenchuan, Li, Mao, Luding, and Xiaojin Counties and Kangding City (Figure 2a). Moderate-suitability areas covered 3.25 × 10^4^ km^2^ (52.17% of total suitable habitat), forming a massive perimeter around high-suitability zones (Figure 2a).

### 2.3. The Future Potential Distribution of P. aquilinum var. latiusculum in the Upper Dadu River–Min River Basin

**Figure 3:**
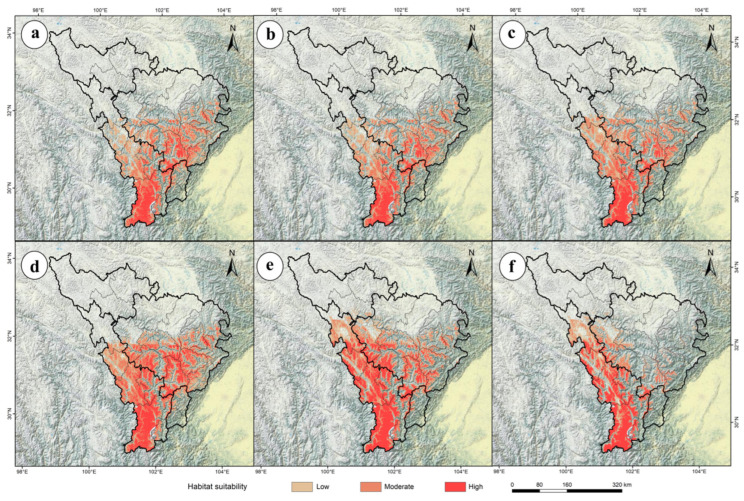
Changes in the potential geographic distribution of *P. aquilinum* var. *latiusculum* in the upper Dadu River–Min River basin under future climate change scenarios. Potential geographic distributions under different climate scenarios: SSP1-2.6 (**a**,**d**), SSP2-4.5 (**b**,**e**), and SSP5-8.5 (**c**,**f**). Potential geographic distributions in different periods: the 2050s (**a**–**c**) and the 2090s (**d**–**f**).

The ensemble model (Ensemble) was used to predict the potential geographic distribution of *P. aquilinum* var. *latiusculum* in the upper Dadu River–Min River basin under three emission scenarios (SSP1-2.6, SSP2-4.5, SSP5-8.5) for 2050 and 2090 (Figure 3), yielding the area of potential geographic distribution under future climate change scenarios (Table 1). The total suitable habitat for *P. aquilinum* var. *latiusculum* increased most significantly under the SSP1-2.6 scenario in the 2090 period, with a 13.00% increase (0.81 × 10^4^ km^2^), while the sharpest decrease occurred under the SSP5-8.5 scenario in the 2090 period, with a 24.07% reduction (1.50 × 10^4^ km^2^) (Table 1). High-suitability areas showed the most significant increase under the SSP5-8.5 scenario in the 2090 period (397.22%, 1.43 × 10^4^ km^2^), whereas the smallest increase occurred under the SSP2-4.5 scenario in the 2050 period (211.11%, 0.76 × 10^4^ km^2^) (Table 1). Moderate-suitability areas rebounded most notably under the SSP1-2.6 scenario in the 2090 period, with the decline narrowing to 11.08% (0.36 × 10^4^ km^2^), while the most severe reduction occurred under the SSP5-8.5 scenario in the 2090 period (58.46%, 1.90 × 10^4^ km^2^) (Table 1). Low-suitability areas experienced the most significant decline under the SSP5-8.5 scenario in the 2090 period (39.31%, 1.03 × 10^4^ km^2^), with the smallest reduction under the SSP2-4.5 scenario in the 2050 period (14.50%, 0.38 × 10^4^ km^2^) (Table 1). These results indicate complex trends under climate change, where high-suitability areas increase concurrently with decreases in moderate- and low-suitability areas for *P. aquilinum* var. *latiusculum*.

**Figure 4:**
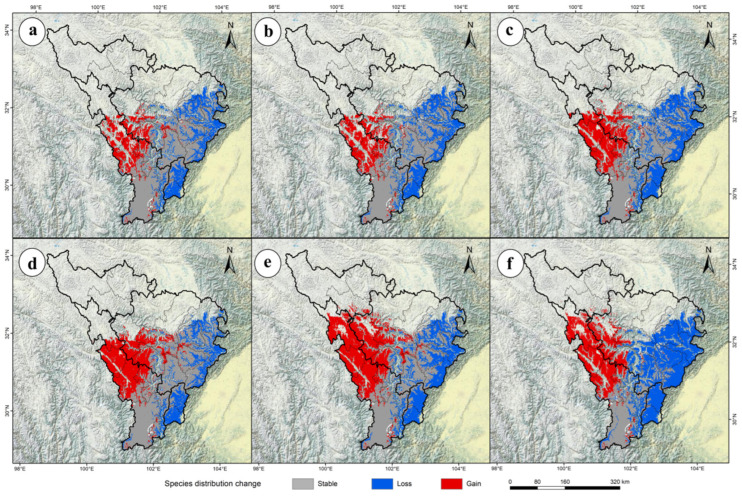
Changes in the potential geographic distribution of *P. aquilinum* var. *latiusculum* in the upper Dadu River–Min River basin under climate change. Potential geographic distribution changes under different climate scenarios: SSP1-2.6 (**a**,**d**), SSP2-4.5 (**b**,**e**), and SSP5-8.5 (**c**,**f**). Changes in potential geographic distribution across different periods: the 2050s (**a**–**c**) and the 2090s (**d**–**f**).

Using the ensemble model (Ensemble), the potential geographic distribution of *P. aquilinum* var. *latiusculum* in the upper Dadu River–Min River basin was predicted under three emission scenarios (SSP1-2.6, SSP2-4.5, SSP5-8.5) for 2050 and 2090 (Figure 3). Spatial overlay analysis of the results in ArcGIS revealed changes in the potential distribution of *P. aquilinum* var. *latiusculum* under future climate change scenarios (Figure 4). Compared to the current period, the area of suitable habitat for *P. aquilinum* var. *latiusculum* is projected to contract in the future (Figure 4). The SSP5-8.5 scenario exhibited the most pronounced trends in both habitat expansion and contraction. In the 2050 period, the most severe contraction occurred under SSP5-8.5, with a 42.09% reduction (2.62 × 10^4^ km^2^) (Table 2). By the 2090 period, contraction under SSP5-8.5 became even more severe, reaching 65.51% (4.08 × 10^4^ km^2^) (Table 2). Conversely, the largest expansion under SSP5-8.5 occurred in the 2050 period (32.65%, 2.03 × 10^4^ km^2^), and further increased to 41.47% (2.58 × 10^4^ km^2^) in the 2090 period (Table 2). These findings indicate that the distribution range of *P. aquilinum* var. *latiusculum* undergoes drastic habitat changes and faces significant stress under high-emission scenarios.

### 2.4. The Movement Trajectory of the Centroid of Suitable Habitats Under Future Climate Change

Based on the centroid of suitable habitats for *P. aquilinum* var. *latiusculum* under current climatic conditions and future climate change scenarios, this study reveals the movement trajectory and trends of its potential suitable habitats. The centroid of the suitable habitat for *P. aquilinum* var. *latiusculum* in the upper Dadu River–Minjiang River basin under the current climate is located at 102.1547° E/31.3252° N (Figure 2b). Under the SSP126 scenario, the centroid of the suitable habitat first moves 4.61 km northwestward to 102.1202° E, 31.3543° N by the 2050s, then shifts 5.96 km northeastward to 102.1750° E, 31.3803° N by the 2090s (Figure 2b). Under the SSP245 scenario, the centroid first moves 3.84 km northwestward to 102.1378° E, 31.3566° N by the 2050s, then continues moving 25.68 km northwestward to 101.9925° E, 31.5518° N by the 2090s (Figure 1b). Under the SSP585 scenario, the centroid first moves 20.02 km northwestward to 102.1105° E, 31.5017° N by the 2050s, then shifts another 19.98 km northwestward to 102.0193° E, 31.6642° N by the 2090s (Figure 2b). Overall, from the baseline climate to the 2050s and 2090s, the centroid of suitable habitats for *P. aquilinum* var. *latiusculum* exhibits the following trends: Under the low-emission (SSP126) scenario, it first moves northwestward toward higher latitudes, then shifts northeastward. Under medium- and high-emission (SSP245, SSP585) scenarios, it consistently moves northwestward toward higher latitudes.

### 2.5. Analysis of Niche Changes in Future Periods

**Figure 5:**
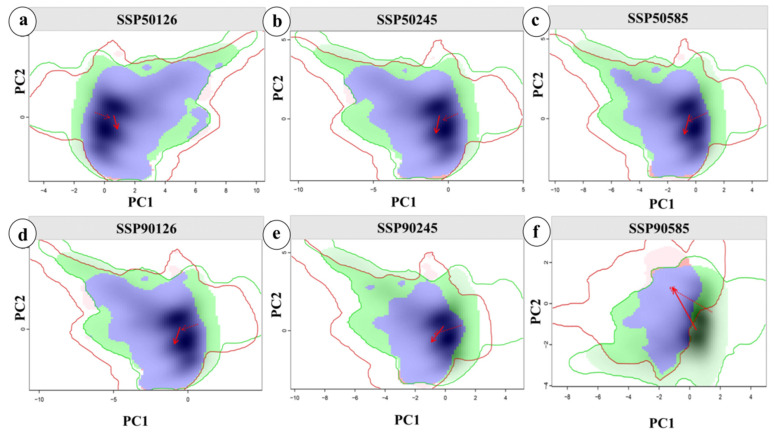
Niche changes in *P. aquilinum* var. *latiusculum* under climate change. Niche shifts under different climate scenarios: SSP1-2.6 (**a**,**d**), SSP2-4.5 (**b**,**e**), and SSP5-8.5 (**c**,**f**). Niche changes across different periods: the 2050s (**a**–**c**) and the 2090s (**d**–**f**). Green and red shading indicate the density of species occurrence in current and future scenarios, respectively, with blue representing overlap. The solid and dashed lines denote 100% and 50% of available environmental space, respectively. The red arrows illustrate how the climatic niche centroid (solid line) and background range centroid (dashed line) shift between these ranges.

This study quantitatively analyzed the niche differentiation and environmental drivers of *P. aquilinum* var. *latiusculum* in the upper Dadu River–Min River basin under three emission scenarios (SSP1-2.6, SSP2-4.5, and SSP5-8.5) for 2050 and 2090. Using distribution points and climate data under different climate backgrounds, the *ecospat* package was employed to calculate niche overlap rates and visualize niche changes for important wild herbivorous resources in the study area (Figure 5). As shown in Figure 5, changes in climatic niches under current and future climate conditions align with background climate trends. The niche overlap rate of *P. aquilinum* var. *latiusculum* significantly decreased under the SSP5-8.5 scenario, with D values of 0.427 in the 2050s and 0.307 in the 2090s, indicating intensified niche differentiation under high-emission scenarios and potential migration to higher-altitude regions. Principal component analysis (PCA) showed that the first two principal components explained 68.69–72.10% of the variance in environmental factors (PC1: 51.53–53.31%; PC2: 17.16–18.79%). The coefficients of variation in temperature seasonality, the annual temperature range, and the minimum temperature of the coldest month identified these as the primary drivers of niche changes. The future climatic niche center will shift toward areas with higher annual temperature ranges and temperature seasonality coefficients.

### 2.6. Delineation of Potential Cultivation Areas for P. aquilinum var. latiusculum in Different Periods

According to the Akaike Information Criterion (AIC), among the seven linear models, the linear model shown in Figure S3c was identified as the optimal model for this study. Figure S3 illustrates a significant positive correlation between habitat suitability and productivity for *P. aquilinum* var. *latiusculum*.

**Figure 6:**
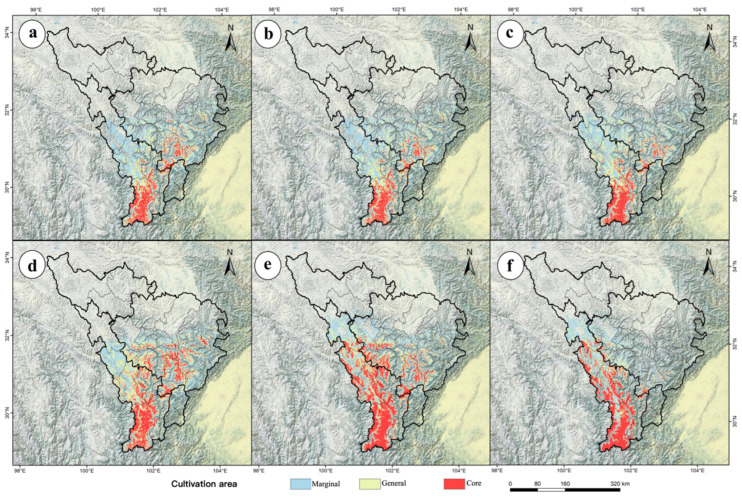
The distribution of the three-level cultivation zones for *P. aquilinum* var. *latiusculum* in the upper Dadu River–Min River basin under future climate scenarios. The three-level cultivation zones under different climate scenarios: SSP1-2.6 (**a**,**d**), SSP2-4.5 (**b**,**e**), and SSP5-8.5 (**c**,**f**). Cultivation zone changes across different periods: the 2050s (**a**–**c**) and the 2090s (**d**–**f**).

Based on the production dynamics model, productivity was categorized into three levels: high productivity (>0.58), medium productivity (0.37–0.58), and low productivity (<0.37). High-productivity areas were designated as core cultivation zones, medium-productivity areas as general cultivation zones, and low-productivity areas as marginal cultivation zones (Figure 6).

Under current climate conditions, the total cultivation area is 6.12 × 10^4^ km^2^, including 0.24 × 10^4^ km^2^ of core cultivation zones and 2.68 × 10^4^ km^2^ of general cultivation zones (Table 3). Current cultivation areas for *P. aquilinum* var. *latiusculum* are primarily concentrated in the entire upper Min River basin and parts of Kangding City, Daofu County, and Danba County in the upper Dadu River basin. Core cultivation zones are predominantly distributed in patchy, fragmented patterns across Tianquan County, Kangding City, Wenchuan County, Xiaojin County, Li County, Mao County, and Heishui County (Figure 2c).

Quantitative analysis of spatiotemporal changes in cultivation areas under different Shared Socioeconomic Pathways (SSPs) (Figure 6) revealed significant shifts across the three cultivation zones compared to the current period. The results show that as time progresses or the intensity of the emission pathway increases, the total cultivation area of *P. aquilinum* var. *latiusculum* exhibits a contraction trend, with core cultivation areas increasing, general cultivation areas fluctuating, and marginal cultivation areas decreasing (Figure 6, Table 3). Climate change induces a northward migration of cultivation areas toward higher latitudes, with core cultivation zones gradually shifting to Kangding City, Danba County, and Daofu County in the upper Dadu River basin (Figure 6). This indicates that under climate change, a large portion of unsuitable areas in the upper Dadu River basin may transform into general or marginal cultivation zones, and even some general/marginal zones could become core cultivation areas. Conversely, many general and marginal cultivation zones in the upper Min River basin may turn unsuitable, with some general/core zones degrading to marginal status (Figure 6). These complex responses highlight that species adaptation to climate change is nonlinear, underscoring the need for adaptive management strategies for *P. aquilinum* var. *latiusculum* in the future.

## 3. Discussion

### 3.1. The Significance of Conducting Distribution Modeling for P. aquilinum var. latiusculum

*P. aquilinum* var. *latiusculum*, a traditionally harvested wild vegetable for residents in the upper Dadu River–Min River basin, serves not only as a supplement to food security in alpine regions, but also as a vital source of nutritional health and economic income [35]. This wild vegetable is renowned for its high nutritional value and medicinal potential [27,29,30,31,32,33]. The biological characteristics and utilization history of *P. aquilinum* var. *latiusculum* in the study area are detailed in Text S2. Field surveys indicate that locals highly favor *P. aquilinum* var. *latiusculum* for its perceived efficacy in reducing phlegm, clearing heat, and calming the mind. The annual collection volume in the upper Min River basin reaches 500 tons, generating an average annual income of CNY 2000 per household. Therefore, understanding changes in its potential distribution and cultivation areas under climate change is crucial for its sustainable utilization in this region. To quantify the potential impacts of climate change on the distribution of *P. aquilinum* var. *latiusculum*, this study implemented a four-phase analytical framework. Set in the context of the upper reaches of the Dadu River–Minjiang River, and focusing on *P. aquilinum* var. *latiusculum*, the research first involved conducting a comparative analysis of 12 common SDMs. Optimal SDMs for the study area were screened via three evaluation metrics. Secondly, environmental variables for modeling were preselected based on collinearity analysis. Thirdly, the optimal SDM was applied to these variables to derive the current and future potential distribution areas, niche change trends, and habitat centroid migration trajectories of *P. aquilinum* var. *latiusculum*. Fourthly, a production dynamics model was constructed using standardized nutritional composition data of *P. aquilinum* var. *latiusculum* to explore its current and future potential natural cultivation regions.

### 3.2. Advantages of Using Ensemble Models in This Study

Niche models utilize known species distribution data and environmental variables to deduce ecological requirements via algorithms, projecting results onto corresponding spatiotemporal regions to simulate actual and potential distributions [39]. Recent studies highlight limitations of single-species distribution models like discriminant analysis or support vector machines, including overfitting, high uncertainty, and data-type dependencies [40,41]. Ensemble models integrate multiple single models to combine their strengths, separate output uncertainties and errors, and map primary trends, thereby mitigating these limitations [40,42]. In this study, the ensemble model was constructed using top-performing single models based on predictive results and evaluation metrics. With mean values of 0.972 for TSS, 0.998 for ROC, and 0.950 for KAPPA, the ensemble model outperformed single models in both scoring and prediction accuracy. This indicates that ensemble models leverage complementarity among single models to reduce bias and uncertainty, yielding more reliable predictions. Notably, the suitable habitats predicted for *P. aquilinum* var. *latiusculum* in the study area align with distribution heatmaps from iPlant (https://www.iplant.cn accessed on 1 April 2025), and the field survey records fall within predicted suitable habitats, further validating the model’s accuracy.

### 3.3. Impacts of Climate Change on P. aquilinum var. latiusculum

Climate change may accelerate species extinction, reduce biodiversity, and weaken regional ecosystems, though some species may develop new physiological traits to adapt [43,44]. Over recent decades, global climate and environmental change research has gained increasing scientific prominence [1,21,23]. Based on environmental factors under three emission scenarios for 2050 and 2090, combined with current climate data, this study predicted the potential geographic distribution of *P. aquilinum* var. *latiusculum*. The results show that under climate change, the potential suitable habitats and cultivation areas generally decline in the upper Dadu River–Min River basin, while high-suitability areas and primary cultivation zones increase drastically. Pauli et al. [45] documented a “mountain-top trap effect” in alpine ecosystems under climate warming, where suitable plant habitats become constrained by summit boundaries. Similarly, *P. aquilinum* var. *latiusculum* in alpine regions faces this effect. Thomas et al.’s research on extinction risks in 20% of global sample areas showed bidirectional impacts of climate warming on species distributions, with neither a risk of universal extinction nor benefits [3]. Parmesan and Yohe [46] noted that climate change may expand the ranges of some species while shrinking those of others, highlighting its dual role as a double-edged sword. For *P. aquilinum* var. *latiusculum* in alpine ecosystems, climate warming induces a polarized pattern, with moderate/low-suitability habitats and low-quality cultivation areas decreasing. In contrast, high-suitability habitats and high-quality cultivation areas increase, rather than a simple linear response occurring.

Regarding niche dynamics, *P. aquilinum* var. *latiusculum* showed decreasing niche overlap between current and future scenarios as climate change intensity increased. The coefficient of variation in temperature seasonality, annual temperature range, and minimum temperature of the coldest month were identified as the primary drivers of niche differentiation (Supplementary Table S1), consistent with the biological characteristics described by Wang [27] and Thepsilvisut et al. [29]. Bai et al. [47] showed that the budding rate and stem emergence period of *P. aquilinum* var. *latiusculum* vary significantly under different environmental conditions. Excessive temperature differences or extremely low temperatures can dramatically affect the yield and distribution of *P. aquilinum* var. *latiusculum*, further validating the accuracy of this study. Rapid temperature changes in high-altitude regions like Tibet and Qinghai [48,49] significantly impact the upper Dadu River–Min River basin, located at the eastern edge of the Qinghai–Tibet Plateau [34], and its flora.

The northward or upward migration of plant habitat centroids under climate change is well-documented. Parmesan and Yohe [46] found that 80% of species migrated poleward or to higher altitudes due to warming, with temperate plants showing particularly strong responses. This pattern is validated across Eurasian ecosystems: Chen et al. [48] observed the northward expansion of European beech (*Fagus sylvatica*) into Scandinavia at 30–50 km per decade, driven by reduced winter cold constraints. Lenoir et al. [50] reported a 29 m per decade upward shift in plant communities across six European mountain ranges, correlated with regional warming rates. In East Asia, Zhu et al. [51] found that 53% of Chinese plant species shifted their climatic niches northward, with alpine/subalpine species in the Hengduan Mountains migrating at a rate of 11 km per decade—faster than low-altitude species—confirming differential climate impacts on mountain ecosystems. The northward and northeastward shift of *P. aquilinum* var. *latiusculum*’s potential habitats in this study aligns with the “climate-driven niche shift” mechanism, supporting the latitude adaptation strategy of Northern Hemisphere plants to warming. The upper reaches of the Dadu River and Minjiang River represent the forest-steppe ecotone of the Qinghai–Tibet Plateau [33,34,35]. This study reveals that the niche of *P. aquilinum* var. *latiusculum* is shifting from the current forest-steppe ecotone to high-altitude grasslands, with a more pronounced migration under high-carbon emission scenarios compared to low-carbon ones. The stability of grassland ecosystems is far lower than that of forest and shrub ecosystems [52]. Therefore, the large-scale development and utilization of *P. aquilinum* var. *latiusculum* in grassland ecosystems would undoubtedly pose a significant challenge to grassland ecological stability and biodiversity. Thus, future development and utilization of *P. aquilinum* var. *latiusculum* in the upper reaches of the Dadu River–Minjiang River should consider its potential migration to high-altitude grasslands under climate change.

### 3.4. Adaptive Management of P. aquilinum var. latiusculum Under Climate Warming

The predicted increase in suitable areas for *P. aquilinum* var. *latiusculum* under climate change offers opportunities for sustainable resource use. The suitability–productivity model developed here delineates potential cultivation zones, providing a scientific basis for future management. Current cultivation areas are concentrated in the upper Min River basin and in the Kangding, Daofu, and Danba Counties in the upper Dadu River basin (Figure 2c), where the hydrothermal conditions match the plants ecological needs. Under the SSP5-8.5 scenario, core cultivation areas are expected to expand to higher altitudes and latitudes by 2090 (Figure 5), suggesting that climate change may enable large-scale cultivation while posing management challenges. As a dual-purpose food–medicine plant [27,29], the predicted expansion of core cultivation areas could facilitate artificial cultivation as an alternative to wild harvesting.

Based on the region’s unique climate, three recommendations are proposed for the sustainable utilization of *P. aquilinum* var. *latiusculum*: (1) Enhance awareness and promote rational exploitation: Increase surveys of wild vegetable resources like *P. aquilinum* var. *latiusculum* to understand their efficacy, distribution, and growth patterns. Adopt a “utilization–protection balance” to ensure ecological sustainability and income growth for local communities. (2) Strengthen systematic research and scientific utilization: Invest in cultivar development through nutritional and health efficacy studies. Develop key cultivation technologies to increase commercial yields and meet growing consumer demand. (3) Amplify promotion and encourage integration with wellness industries: Leverage the region’s status as a core area of the Tibetan–Qiang–Yi Cultural Industry Corridor (State Council Bulletin, https://www.gov.cn accessed on 5 April 2025). Promote wild vegetable products alongside wellness tourism to boost local economies and public health. Prioritize standardized cultivation in current core zones and adopt adaptive management strategies that incorporate dynamic adjustments to cultivation zones.

### 3.5. Research Prospects

This study provides a macro-planning foundation for the sustainable management of *P. aquilinum* var. *latiusculum* resources in alpine valley areas under climate change scenarios, but several aspects require further refinement. First, although the Human Footprint Index was incorporated into the modeling, its contribution rate was not fully quantified due to the spatial characteristic of overall low human activity intensity in the study area. In the future, there is an urgent need to integrate high-spatiotemporal-resolution dynamic data on human activities to construct a “climate–human dual-driving model” for the precise assessment of picking pressure and land use competition effects in hotspot regions, such as Wenchuan County and Kangding City. Second, the synergistic effects of multidimensional influencing mechanisms need to be systematically analyzed. This involves coupling land use change models to simulate habitat fragmentation processes, incorporating community-level socioeconomic driving factors, and quantifying the competition coefficients between *P. aquilinum* var. *latiusculum* and associated plants through field control experiments to improve niche dynamic predictions. Given the inherent unpredictability of future variables, practical applications must comprehensively evaluate these confounding factors within adaptive management frameworks to ensure effective decision-making.

## 4. Materials and Methods

### 4.1. Sample Collection and Species Distribution Records

From July 2022 to May 2024, our research team conducted field surveys across the potential distribution areas of *P. aquilinum* var. *latiusculum* in the upper Dadu River–Min River basin, which yielded 256 validated occurrence records. Using the subset and clean_coordinates functions in the R package CoordinateCleaner(V2.0), we corrected spatial biases in the dataset by retaining only one occurrence point per 1 km × 1 km grid cell. This process resulted in 176 high-quality occurrence points (Figure 2d) [53].

### 4.2. Selection and Processing of Environmental Variables

A total of 41 environmental variables were initially considered, including 19 bioclimatic factors, 16 soil properties, 3 topographic variables, 1 Human Footprint Index, 1 land use type, and 1 Normalized Difference Vegetation Index (NDVI). Current and future climate data were downloaded from the WorldClim database (http://worldclim.org/data/index.html accessed on 1 August 2023) under three Shared Socioeconomic Pathways (SSP1-2.6, SSP2-4.5, and SSP5-8.5), representing scenarios of low, medium, and high greenhouse gas emissions, respectively. Soil and topographic data were sourced from the Harmonized World Soil Database (HWSD) of the Food and Agriculture Organization (FAO) (http://www.fao.org/faostat/en/#data accessed on 1 August 2023). The Human Footprint Index 2009, derived from eight variables (built environments, population density, electrical infrastructure, cropland, pasture, roads, railways, and navigable waterways), was compiled by the NASA Socioeconomic Data and Applications Center (SEDAC). NDVI data, calculated as the difference between near-infrared and red reflectance, were provided by the Land Processes Distributed Active Archive Center (LPDAAC) at the U.S. Geological Survey’s Earth Resources Observation and Science (EROS) Center (http://LPDAAC.usgs.gov accessed on 1 August 2023). Land use data were acquired from the Resource and Environment Data Cloud Platform of the Chinese Academy of Sciences (http://www.resdc.cn/Default.aspx accessed on 1 August 2023).

To mitigate multicollinearity and overfitting in model predictions, we performed variable screening using variance inflation factor (VIF) analysis, principal component analysis (PCA), and Spearman’s correlation tests in R [16]. Variables were retained if they met the following criteria: (1) a pairwise Spearman correlation coefficient < 0.7, (2) VIF < 5, and (3) higher ecological relevance for *Pteridium aquilinum* var. *latiusculum*. This process resulted in the selection of 16 environmental variables (Table S1).

### 4.3. Ensemble Model Construction

Biomod2 was used to develop an ensemble model based on species distribution data and pseudo-absence points. The package generates non-occurrence data (pseudo-absences) from background environmental data using predefined methods. The random command was employed to create 1200 pseudo-absence points for model simulation. The biomod_tuning function optimized model parameters and selected 75% of the dataset for training, with the remaining 25% reserved for validation. Equal weights were assigned to occurrence and pseudo-absence data, and the process was repeated 10 times to produce 100 model simulations. Model accuracy was evaluated using the Receiver Operating Characteristic (ROC), Kappa, and True Skill Statistic (TSS). Single models with TSS ≥ 0.7 were retained, and a weighted average method was applied to construct the ensemble model [10]. A binary threshold (cutoff) of 0/1 was derived from model outputs, where areas below the threshold were classified as unsuitable. Regions above the threshold were divided into three equal suitability tiers: low, moderate, and high. The final classifications were visualized in ArcGIS v10.4.1.

### 4.4. Niche Dynamics Analysis

For current climate conditions, the background point selection area for *Pteridium aquilinum* var. *latiusculum* was defined by a 1-degree buffer around known distribution points. Under future climate scenarios, background areas were delineated using ensemble-modeled suitable habitats. Niche overlap between current and future climates was calculated using the *ecospat* package, which visualized niche shifts and quantified the D statistic (range: 0–1), where 0 indicates no overlap and 1 signifies complete overlap [13,23]. This assessed the impacts of climate change on species niche dynamics.

### 4.5. Centroid Migration Trajectory

Using the SDMTool package (v1.1-21) in R, centroid coordinates of suitable habitats were calculated for different periods and climate scenarios. Longitude/latitude values and migration distances between centroids were computed in ArcGIS, and trajectory visualizations were generated to illustrate centroid shifts.

### 4.6. Establishing Cultivation Productivity–Suitability Relationships

This study employed food science methods to determine the nutritional components (conventional nutrients, bioactive compounds, and amino acid profiles) of *P. aquilinum* var. *latiusculum* across 36 randomly selected distribution sites. An assessment model integrating ecological suitability and nutritional components was developed to evaluate the cultivation productivity of *P. aquilinum* var. *latiusculum*. The assessment model is presented below.

(1) P=S+N

To evaluate the relationship between the cultivation productivity and environmental suitability of *P. aquilinum* var. *latiusculum*, the ecological suitability value (*S*) was derived from the presence probability values output by the species distribution model, with suitability data for each cultivation area extracted via spatial interpolation and weighted accordingly. Nutritional components (N) had their indicator weights determined by the entropy weight method. The types of *P. aquilinum* var. *latiusculum* nutritional components, weight ratios, and rationales are detailed in Text S1 and Table S4. After standardizing each indicator (Table S2) [54], a weighted summation was performed using the following formula:

(2) $$N=\sum_{i=1}^{4}w_{i}\cdot X_{i}^{norm}$$

Model validation utilized the ggtrendline package in R (v4.1.2) to fit quantitative relationships between cultivation productivity and ecological suitability using seven types of nonlinear regression models (Table S3) [53]. The optimal model was selected based on the Akaike Information Criterion (AIC), with ΔAIC < 2. The potential cultivation areas of *P. aquilinum* var. *latiusculum* under current and future climate conditions were then predicted using this optimal model [55].

## 5. Conclusions

This study employed an ensemble modeling approach to systematically evaluate the impacts of climate change on the potential distribution and cultivation areas of *P. aquilinum* var. *latiusculum* in the upper Dadu River–Min River basin, revealing complex response mechanisms in its suitable habitats and cultivation patterns. The results indicate that current high-suitability areas and core cultivation zones for *P. aquilinum* var. *latiusculum* are fragmented across low-altitude river valleys, such as those in the Wenchuan and Li Counties. Under climate warming, both potential distribution and cultivation areas exhibit a polarized pattern of “expansion in high-quality zones and contraction in moderate/low-quality zones,” accompanied by gradual shifts in climatic niches. Adaptive management strategies are recommended for sustainable utilization, involving the standardization of cultivation practices in existing core zones to meet market demands while dynamically adjusting cultivation areas in response to changing climate suitability. This research not only provides decision-making support for the sustainable use of *P. aquilinum* var. *latiusculum* resources, but also offers insights for biodiversity conservation and livelihood adaptation in alpine valley regions under global change.

## Figures and Tables

**Table 1:** Potential suitable areas for *P. aquilinum* var. *latiusculum* in different periods (10^4^ km^2^).

Period	Climate Scenario	Low Suitability Zone	Moderate Suitability Zone	High Suitability Zone	Total Suitability Zone
Current		2.62	3.25	0.36	6.23
2050	SSP126	2.29	2.18	1.19	5.66
2050	SSP245	2.24	2.05	1.12	5.41
2050	SSP585	2.11	2.31	1.22	5.64
2090	SSP126	2.09	2.89	2.06	7.04
2090	SSP245	2.08	2.42	2.74	7.24
2090	SSP585	1.59	1.35	1.79	4.73

**Table 2:** Changes in suitable habitat areas for *P. aquilinum* var. *latiusculum* under climate change scenarios (10^4^ km^2^).

Period	Climate Scenario	Habitat Area	Loss	Stable	Gain
2050	SSP126	5.66	2.04	4.19	1.48
2050	SSP245	5.41	2.29	3.94	1.47
2050	SSP585	5.64	2.62	3.61	2.03
2090	SSP126	7.04	1.82	4.41	2.63
2090	SSP245	7.24	2.55	3.68	3.56
2090	SSP585	4.73	4.08	2.15	2.58

**Table 3:** Areas of potential cultivation zones for *P. aquilinum* var. *latiusculum* in different periods (10^4^ km^2^).

Period	Climate Scenario	Marginal Cultivation	General Cultivation	Core Cultivation	Total Cultivation
Current		3.20	2.68	0.24	6.12
2050	SSP126	2.55	1.95	0.98	5.48
2050	SSP245	2.49	1.84	0.91	5.24
2050	SSP585	2.46	2.05	1.01	5.52
2090	SSP126	2.43	2.77	1.71	6.91
2090	SSP245	2.31	2.38	2.39	7.08
2090	SSP585	1.68	1.28	1.63	4.59

## Data Availability

Data will be made available on request.

## Supplementary Materials

The following supporting information can be downloaded at https://www.mdpi.com/article/10.3390/plants14142123/s1: Figure S1: (a) Field habitat of *P. aquilinum* var. *latiusculum*; (b–d) Three processing and consumption methods of *P. aquilinum* var. *latiusculum*: (b) Blanched and served with cold dressing; (c) Blanched and stir-fried with meat; (d) Dried, blanched, and served with cold dressing; Figure S2: Potential distribution of *P. aquilinum* var. *latiusculum* in the upper Dadu River–Minjiang River basin based on multi-model predictions: (a) ANN model, (b) GTA model, (c) FDA model, (d) GAM model, (e) GBM model, (f) GLM model, (g) MARS model, (h) MaxEnt model, (i) RF model, (j) SER model, (k) XGBOOST model, (l) Ensemble model. Figure S3: Relationships between suitability and productivity for *P. aquilinum* var. *latiusculum*. (a) exp2P model, (b) exp3P model, (c) line2P model, (d) line3P model, (e) log2P model, (f) power2P model, (g) power3P model; Text S1: Nutritional Component Types, Weight Proportions, and Rationale for Setting Weights; Text S2: Biological Characteristics and Utilization History of *P. aquilinum* var. *latiusculum* in the Study Area. Table S1: 16 environmental variables involved in modeling; Table S2: Seven model types used for modeling the relationship between productivity and suitability; Table S3: Standardized results of various indicators for *P. aquilinum* var. *Latiusculum*; Table S4: Types of Nutritional Components and Assigned Weight Proportions for *P. aquilinum* var. *Latiusculum.*
