# Supplementary material for: Modeling the Future of a Wild Edible Fern Under Climate Change: Distribution and Cultivation Zones of Pteridium aquilinum var. latiusculum in the Dadu–Min River Region
Source: Plants (Basel). 2025 Jul 9;14(14):2123. doi: 10.3390/plants14142123 (PMC12299327; doi:10.3390/plants14142123)
Supplement: Supplementary file 1 [file plants-14-02123-s001.zip › plants-3709024-supplementary.pdf]

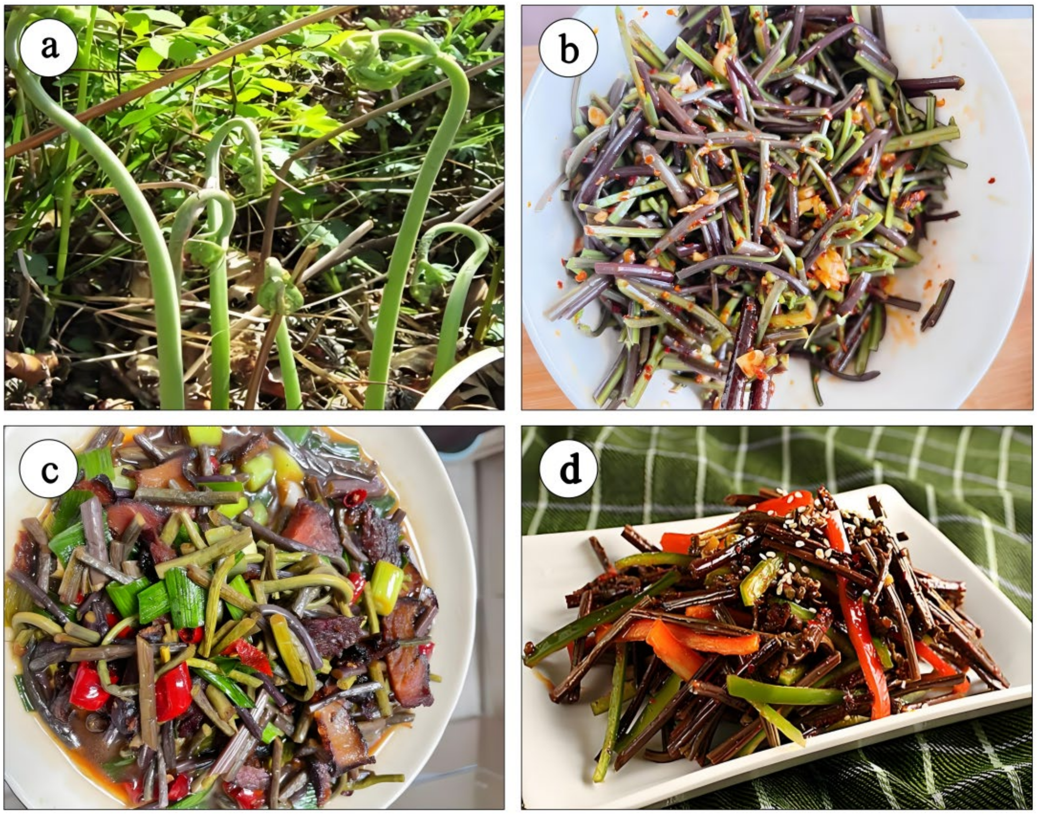

Figure S1. (a) Field habitat of *P. aquilinum* var. *latiusculum*; (b–d) Three processing and consumption methods of *P. aquilinum* var. *latiusculum*: (b) Blanched and served with cold dressing; (c) Blanched and stir-fried with meat; (d) Dried, blanched, and served with cold dressing.

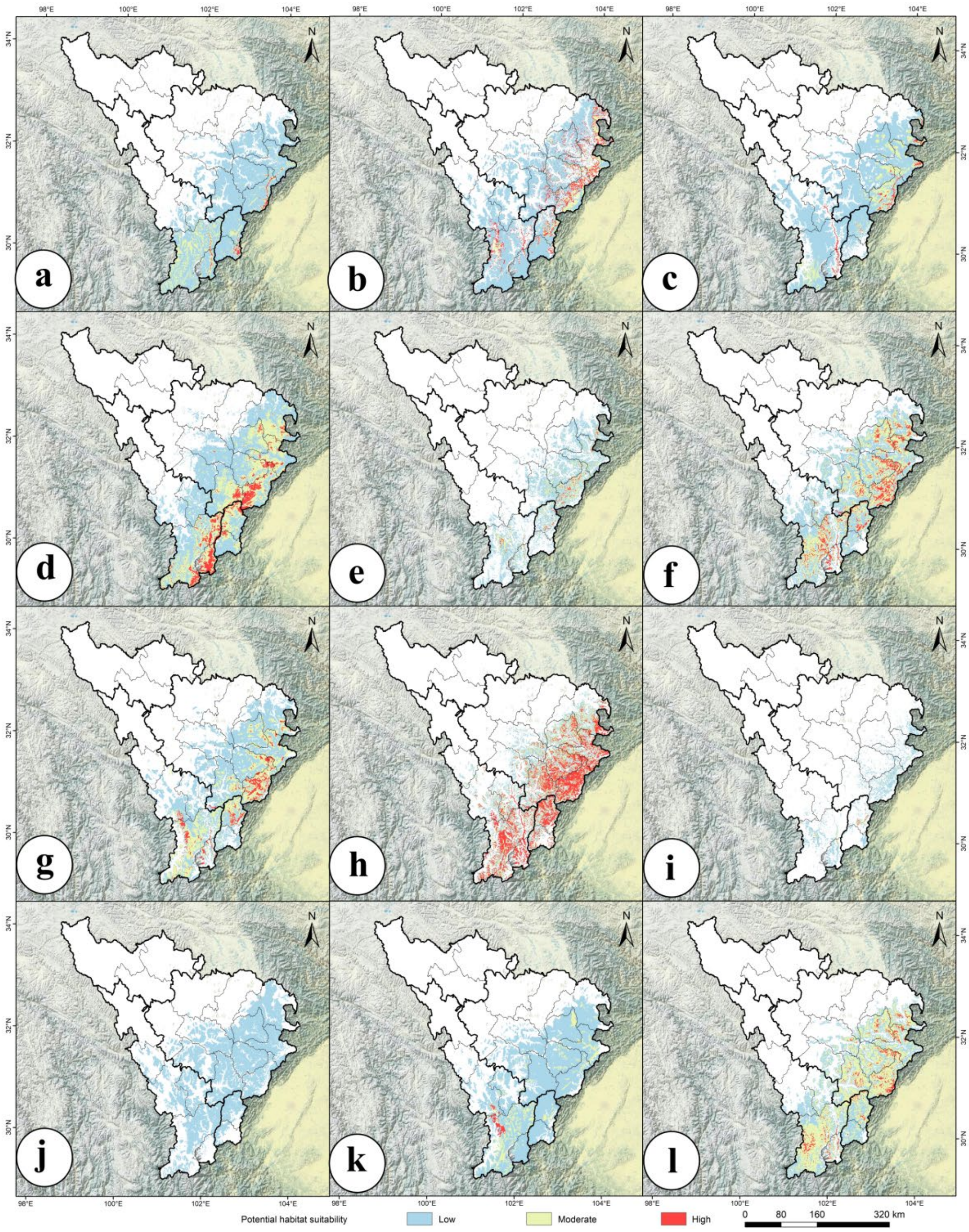

Figure S2. Potential distribution of *P. aquilinum* var. *latiusculum* in the upper Dadu River–Minjiang River basin based on multi-model predictions: (a) ANN model, (b) GTA model, (c) FDA model, (d) GAM model, (e) GBM model, (f) GLM model, (g) MARS model, (h) MaxEnt model, (i) RF model, (j) SER model, (k) XGBOOST model, (l) Ensemble model.

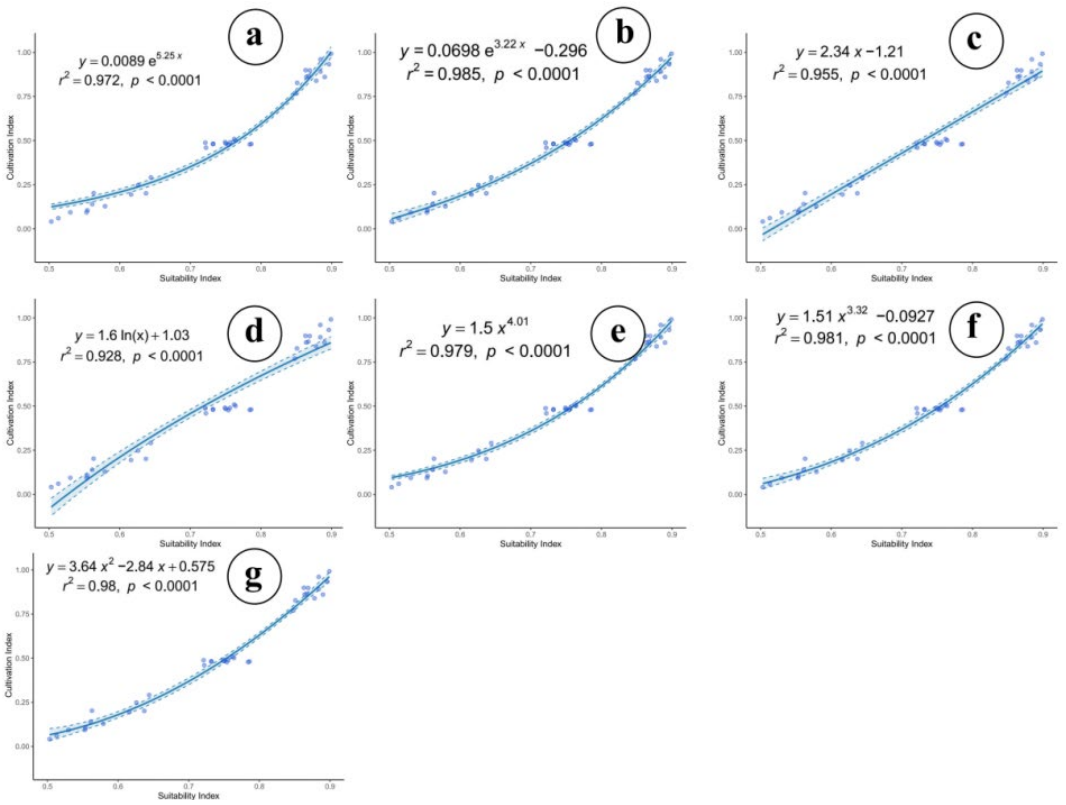

Figure S3. Relationships between suitability and productivity for *P. aquilinum* var. *latiusculum*. (a) exp2P model, (b) exp3P model, (c) line2P model, (d) line3P model, (e) log2P model, (f) power2P model, (g) power3P model.

Text S1. Nutritional Component Types, Weight Proportions, and Rationale for Setting Weights

This study measured routine nutritional components, bioactive substances, and amino acid compositions of *P. aquilinum* var. *latiusculum*. After multiple discussions by experts from the Sichuan Provincial Key Laboratory of Ecology and the Sichuan Provincial Engineering Center for Disaster Prevention and Mitigation in Universities, it was determined that weights should be assigned to each component based on nutritional function, health value, and environmental sensitivity (Table S4).

The total weight was set at 100%. Routine nutrients and bioactive components accounted for 75%, with the following allocations:

- **Protein (12%):** As a core indicator of growth and quality, significantly regulated by nitrogen utilization efficiency.
- **Dietary fiber (10%):** Reflects soil and water conditions through cell wall component regulation and supports gut health functions.
- **Fat (7%), crude fiber (5%), and ash content (1%):** Allocated sequentially based on energy density, stress resistance, and mineral content.
- **Starch (15%):** A core component of carbohydrate metabolism, whose synthesis is regulated by light and temperature via key enzymes such as ADP-glucose pyrophosphorylase.
- **Total flavonoids (12%):** Enhance antioxidant capacity via UV-B-induced

phenylpropanoid metabolism.

- **Total saponins (8%) and tannins (5%):** Reflect immune potential and stress responses, respectively.

Amino acid compositions accounted for 25%, with the following allocations:

- **Essential amino acids (12%):** Each essential amino acid accounts for 1.5%, dependent on nitrogen metabolism efficiency.
- **Conditionally essential amino acids (5%):** Arginine (2%, involved in salt stress response), cysteine (1.5%, reflecting sulfur metabolism levels), and tyrosine (1.5%, indicating secondary metabolism regulation capacity).
- **Glutamic acid (4%):** Serves as a nitrogen metabolism hub in salt stress regulation.
- **Other non-essential amino acids (4%):** Proline (2%, responding to drought via osmotic regulation), with the remaining amino acids each accounting for 0.5%.

Text S2. Biological Characteristics and Utilization History of *P. aquilinum* var. *latiusculum* in the Study Area

An important wild vegetable, *P. aquilinum var. latiusculum*, is widely distributed in moist hillsides, forest edges, and shrublands in the upper reaches of the Dadu River–Minjiang River. The plant can grow up to 1 meter in height. Its rhizomes are long and creeping, densely covered with rust-colored pubescence that gradually sheds over time. *P. aquilinum* var. *latiusculum* has well-developed underground rhizomes that spread prostrate, enabling it to form communities in the complex alpine gorge terrain of western Sichuan. It is one of the first tender wild vegetables to sprout on forest floors in spring.

Ethnic groups such as the Tibetan, Yi, and Qiang in the upper reaches of the Dadu River–Minjiang River have a long history of utilizing *P. aquilinum* var. *latiusculum*. The core of this tradition is its edibility: each spring, local residents harvest large quantities of tender fiddlehead leaves. To remove their mild toxicity and bitter taste, traditional processing techniques such as blanching (boiling in water) or soaking in ash water (plant ash solution) are used. Afterwards, the leaves can be served cold, stir-fried, made into soup, or dried/salted for long-term storage, serving as an important supplement during the spring vegetable shortage. In addition to young leaves, its starch-rich rhizomes were historically dug up, mashed, filtered, and precipitated to make "fern root starch," a famine relief food. In traditional medicine, *P. aquilinum* var. *latiusculum* is believed to have effects such as clearing heat, relaxing the intestines, descending qi, and resolving phlegm.

Table S1. 16 environmental variables involved in modeling.

Environment variable	Abbreviation	Unit	Contribution Rate (%)
Maximum temperature of the warmest month	bio5	°C	15.27
Annual mean temperature	bio1	°C	5.59
Annual precipitation	bio12	mm	13.24
Precipitation of the driest month	bio14	mm	0.15
Precipitation seasonality (Coefficient of Variation)	bio15	C of V	0.38
Temperature seasonality	bio4	C of V	18.27

(Coefficient of Variation)			
Minimum temperature of the coldest month	bio6	°C	31.51
Annual temperature range	bio7	°C	8.5
Seasonal dry matter production	dmps	g/m²/season	3.84
Elevation	elev	m	0.63
Ecological footprint	footprint	gha	0.46
Gross primary productivity	gpp	g C/m²/year	0.09
Land cover	landcover	/	0.08
Gravel content	t_gravel		0.87
Soil organic carbon content	t_oc	%	1.11
Soil pH (water extract)	t_ph_h2o	/	0.01

Table S2. Seven model types used for modeling the relationship between productivity and suitability.

Model code	Model type
exp2P	y=a*exp(b*x)
exp3P	y=a*exp(b*x)+c
line2P	y = a*x + b
line3P	y=a*x^2+b*x+c
log2P	y=a*ln(x)+b
power2P	y=a*x^b
power3P	y=a*x^b+c

Table S3. Standardized results of various indicators for *P. aquilinum* var. *latiusculum*

			**Conventional nutritional components**						**Active substance components**			**Amino acid components**																
Longitude	Latitude	*S*uitability	CFT	CFR	CAH	CPN	DFR	STH	SAN	FLD	TAN	ASP	THR	SER	GLU	GLY	ALA	PRO	CYS	VAL	MET	ILE	LEU	TYR	PHE	HIS	LYS	ARG
101.822	30.007	0.99	7.00	5.00	1.00	12.00	10.00	15.00	8.00	12.00	5.00	0.48	1.50	0.50	4.00	0.50	0.50	2.00	0.92	1.46	1.50	1.50	1.46	1.38	1.50	1.50	1.50	2.00
103.458	31.303	0.96	6.82	4.88	0.99	11.73	9.77	14.80	7.43	11.78	4.81	0.47	1.45	0.48	3.88	0.49	0.48	1.92	1.04	1.28	1.43	1.46	1.40	1.25	1.38	1.40	1.46	1.76
102.831	30.637	0.93	6.64	4.76	0.98	11.45	9.55	14.60	6.86	11.33	4.72	0.46	1.40	0.47	3.76	0.47	0.47	1.85	1.15	1.31	1.36	1.39	1.36	1.19	1.30	1.29	1.43	1.71
102.722	31.241	0.90	6.46	4.51	0.98	10.91	9.32	14.20	6.29	11.22	4.53	0.45	1.30	0.45	3.52	0.46	0.44	1.69	1.15	1.43	1.29	1.20	1.30	1.38	1.38	1.40	1.13	1.51
102.587	31.252	0.90	6.75	4.80	0.98	10.58	9.13	14.10	6.29	11.69	4.62	0.44	1.35	0.42	3.44	0.40	0.49	1.69	1.14	1.33	1.24	1.22	1.33	1.18	1.20	1.17	1.35	1.43
102.138	29.978	0.89	6.53	4.69	0.97	11.35	8.90	14.54	5.71	10.98	4.06	0.46	1.25	0.44	3.42	0.42	0.43	1.62	1.27	1.37	1.14	1.28	1.38	1.31	1.34	1.29	1.28	1.66
103.355	32.115	0.87	6.31	4.45	0.97	9.76	8.56	13.64	5.71	10.62	4.91	0.43	1.08	0.41	3.17	0.41	0.41	1.35	1.36	1.50	1.21	1.41	1.50	1.43	1.41	1.35	1.38	1.77
103.805	32.378	0.86	6.10	4.39	0.97	10.64	8.64	13.80	5.71	10.67	4.43	0.44	1.20	0.43	3.27	0.43	0.42	1.54	1.27	1.34	1.07	1.24	1.40	1.25	1.26	1.24	1.32	1.61
102.574	31.876	0.86	6.28	4.32	0.96	10.36	8.41	13.70	5.71	10.44	4.34	0.43	1.25	0.42	3.39	0.42	0.43	1.62	1.27	1.37	1.21	1.28	1.40	1.31	1.34	1.34	1.35	1.66
103.735	31.925	0.84	5.88	4.27	0.97	10.09	8.16	13.38	5.14	10.11	4.25	0.50	1.33	0.49	3.65	0.45	0.40	1.80	1.30	1.27	0.98	1.20	1.32	1.33	1.41	1.36	1.11	1.78
103.131	31.144	0.83	5.92	4.15	0.96	9.82	7.95	13.00	5.14	10.22	4.15	0.43	1.15	0.40	3.15	0.39	0.40	1.31	1.38	1.40	1.14	1.31	1.44	1.50	1.46	1.45	1.21	1.85
103.167	30.969	0.77	5.74	3.98	0.95	9.27	7.50	12.60	4.57	9.78	4.06	0.41	1.05	0.39	2.91	0.38	0.37	1.15	1.50	1.24	0.93	1.13	1.34	1.13	1.18	1.14	0.95	1.27
101.572	30.099	0.51	2.02	2.27	0.40	7.31	4.00	8.06	2.86	6.93	3.77	0.34	0.85	0.26	2.11	0.28	0.24	1.00	0.69	0.79	0.64	0.90	1.00	0.94	0.85	1.09	0.59	0.73
103.529	32.144	0.50	3.68	1.44	0.47	5.67	5.04	9.46	4.00	3.38	4.25	0.33	0.90	0.22	2.42	0.22	0.19	0.92	0.81	0.73	0.79	0.75	0.98	0.75	0.69	1.03	0.48	0.54
103.061	32.034	0.49	2.85	2.21	0.35	6.55	4.09	7.60	2.86	6.22	3.58	0.36	0.90	0.25	1.94	0.29	0.23	1.00	0.58	0.76	0.64	0.86	0.98	0.81	0.81	1.09	0.62	0.68
102.351	31.523	0.49	3.03	1.85	0.59	6.00	4.77	8.60	3.43	4.67	3.30	0.32	0.75	0.24	2.06	0.28	0.23	0.85	0.81	0.79	0.79	0.83	0.96	0.88	0.77	0.98	0.66	0.59
102.975	31.309	0.49	3.93	2.82	0.27	3.27	6.36	5.20	1.14	5.56	3.68	0.40	1.00	0.30	2.55	0.33	0.27	1.31	0.92	0.91	1.00	1.05	1.10	1.19	1.09	1.29	0.84	1.02
103.585	31.485	0.48	3.21	1.60	0.47	5.45	5.23	9.00	4.00	3.56	3.77	0.31	0.80	0.23	2.18	0.24	0.20	0.92	0.69	0.70	0.71	0.79	0.94	0.69	0.65	1.03	0.51	0.49
102.561	31.749	0.48	3.39	1.97	0.55	4.91	5.00	8.20	3.43	4.89	3.21	0.33	0.70	0.26	2.11	0.26	0.21	0.92	0.81	0.82	0.79	0.90	0.92	0.75	0.73	0.98	0.59	0.63
101.713	30.049	0.48	3.24	2.11	0.94	5.69	4.95	7.91	2.75	5.13	3.04	0.31	0.65	0.25	1.89	0.26	0.22	0.77	0.92	0.76	0.71	0.86	0.90	0.81	0.81	0.93	0.62	0.63
102.269	31.182	0.48	3.75	2.57	0.43	3.82	5.91	5.60	1.71	5.33	3.11	0.38	0.95	0.29	2.42	0.32	0.25	1.23	1.04	0.88	0.93	1.01	1.04	1.06	0.97	1.24	0.77	0.93
101.574	30.204	0.48	4.04	2.62	0.47	4.04	5.80	6.26	1.71	5.09	2.92	0.37	0.80	0.28	2.18	0.29	0.25	1.15	1.04	0.85	0.93	0.98	1.06	1.00	0.93	1.19	0.70	0.88
101.739	29.936	0.48	3.57	2.33	0.51	4.36	5.45	6.60	2.29	5.11	3.02	0.34	0.85	0.27	2.30	0.30	0.24	1.08	0.92	0.82	0.86	0.94	1.00	0.94	0.89	1.14	0.73	0.83
102.275	31.354	0.46	2.67	1.36	0.31	6.00	3.18	9.60	4.57	2.89	3.96	0.30	0.75	0.21	2.06	0.21	0.18	0.85	0.69	0.67	0.86	0.71	0.96	0.63	0.57	0.93	0.40	0.44
103.871	31.692	0.29	1.23	0.88	0.06	2.73	2.50	5.20	2.86	2.67	2.36	0.12	0.45	0.09	0.73	0.11	0.11	0.46	0.81	0.43	1.07	0.38	0.40	0.75	0.61	1.03	0.59	0.54
103.706	32.029	0.25	1.41	0.75	0.08	2.18	2.27	4.60	2.29	2.44	1.89	0.10	0.40	0.08	0.61	0.08	0.09	0.38	0.69	0.36	0.79	0.34	0.36	0.56	0.41	0.88	0.40	0.39
103.755	32.088	0.20	1.05	0.63	0.16	1.64	2.05	4.00	1.71	2.22	1.42	0.08	0.35	0.07	0.36	0.05	0.07	0.23	0.46	0.21	0.71	0.30	0.30	0.44	0.32	0.78	0.33	0.29
101.644	30.305	0.20	1.66	0.85	0.14	1.80	1.90	3.62	1.14	1.98	1.51	0.10	0.30	0.06	0.56	0.07	0.08	0.31	0.58	0.33	0.57	0.26	0.30	0.50	0.28	0.67	0.29	0.24
103.541	31.878	0.19	1.26	0.53	0.37	2.51	1.38	3.34	1.71	1.60	2.36	0.06	0.35	0.04	0.48	0.01	0.06	0.15	0.35	0.27	0.64	0.23	0.26	0.38	0.24	0.57	0.18	0.10
101.409	30.557	0.14	0.87	0.39	0.27	1.36	1.14	1.60	1.14	1.33	1.70	0.07	0.30	0.05	0.41	0.04	0.04	0.08	0.35	0.15	0.57	0.26	0.24	0.31	0.24	0.62	0.26	0.20
103.771	32.171	0.13	0.69	0.27	0.20	1.09	1.36	2.60	1.14	1.11	0.94	0.04	0.25	0.01	0.32	0.03	0.03	0.15	0.23	0.12	0.50	0.19	0.20	0.25	0.20	0.52	0.22	0.15
102.895	31.267	0.10	0.51	0.20	0.23	0.82	0.91	2.20	0.57	0.89	1.13	0.04	0.20	0.04	0.29	0.03	0.03	0.23	0.23	0.09	0.43	0.15	0.18	0.19	0.16	0.47	0.18	0.10
101.781	30.531	0.09	0.12	0.02	0.52	1.31	0.29	1.16	0.46	0.31	1.98	0.08	0.08	0.08	0.36	0.03	0.02	0.38	0.23	0.18	0.29	0.08	0.16	0.31	0.10	0.52	0.07	0.20
101.893	30.531	0.09	0.76	0.15	0.14	0.82	1.01	2.08	0.57	0.80	0.38	0.03	0.20	0.01	0.24	0.04	0.05	0.08	0.12	0.09	0.43	0.15	0.24	0.19	0.16	0.41	0.15	0.05
101.521	30.302	0.06	0.32	0.10	0.43	0.55	0.45	0.60	0.57	0.44	0.47	0.01	0.15	0.03	0.12	0.01	0.01	0.31	0.12	0.06	0.36	0.11	0.10	0.13	0.12	0.26	0.11	0.10

101.543	30.683	0.04	0.14	0.03	0.47	0.44	0.23	0.48	0.46	0.25	0.30	0.01	0.10	0.02	0.10	0.01	0.01	0.06	0.09	0.05	0.23	0.06	0.08	0.10	0.10	0.21	0.06	0.04

Table S4. Types of Nutritional Components and Assigned Weight Proportions for *P. aquilinum* var. *latiusculum*

No.	Nutritional Component	Weight (%)	No.	Nutritional Component	Weight (%)
1	Ash content	1	14	Tyrosine	1.5
2	Protein	12	15	Arginine	2
3	Dietary fiber	10	16	Glutamic acid	4
4	Fat	7	17	Aspartic acid	0.5
5	Crude fiber	5	18	Serine	0.5
6	Starch	15	19	Glycine	0.5
7	Total flavonoids	12	20	Alanine	0.5
8	Total saponins	8	21	Proline	2
9	Tannins	5	22	Cysteine	1.5
10	Threonine	1.5	23	Leucine	1.5
11	Valine	1.5	24	Phenylalanine	1.5
12	Methionine	1.5	25	Lysine	1.5
13	Isoleucine	1.5	26	Histidine	1.5
